# Control of Enzymatic Browning in Strawberry, Apple, and Pear by Physical Food Preservation Methods: Comparing Ultrasound and High-Pressure Inactivation of Polyphenoloxidase

**DOI:** 10.3390/foods11131942

**Published:** 2022-06-29

**Authors:** Filipa Vinagre Marques Silva, Alifdalino Sulaiman

**Affiliations:** 1 LEAF, Associate Laboratory Terra, Instituto Superior de Agronomia, Universidade de Lisboa, Tapada da Ajuda, 1349-017 Lisboa, Portugal; 2 Department of Process and Food Engineering, Universiti Putra Malaysia, Seri Kembangan 43300, Selangor, Malaysia; alifdalino@upm.edu.my

**Keywords:** fruit puree, clean label, sustainable, sonication, thermosonication, high pressure processing, HPP, HPTP, thermal processing, pasteurization, enzyme, PPO, kinetics, enzyme resistance

## Abstract

Polyphenoloxidase (PPO) enzyme can be found in fruits, vegetables and crustaceans. Its activity, promoted by oxygen, causes food browning with subsequent loss of quality and limited shelf life. Foods are pasteurized with conventional and novel physical methods to inactivate spoilage enzymes, thus avoiding the addition of unhealthy chemical preservatives. Ultrasound and high- pressure processing (HPP) are non-thermal technologies capable of retaining vitamins, bioactives and sensory components of fresh fruits. Enzyme residual activity vs. processing time were plotted for strawberry, apple, and pear purees subjected to thermosonication (1.3 W/g—71 °C), HPP-thermal (600 MPa—71 °C) and heat treatment alone at 71 °C. The PPO residual activities after treatments were highly variable. TS was the most effective for inactivating PPO, followed by thermal processing. HPP-thermal did not improve the inactivation compared with thermal treatment at 71 °C. The resistance of the three fruits’ PPOs exhibited the same pattern for the three technologies: pear PPO was the most resistant enzyme, followed by apple PPO and, lastly, strawberry PPO. However, the resistance of the three PPOs to TS was lower and very similar. Given the huge variability of PPO resistance, it is important to run inactivation tests for different fruits/cultivars. The results can assist manufacturers to avoid browning during processing, storage and distribution of fruit purees, juices and concentrates.

## 1. Introduction

### 1.1. Enzymatic Browning by Polyphenoloxidase (PPO)

It is well known that fruits, vegetables and some crustaceous seafood (e.g., lobster, shrimp) experience enzymatic browning when tissues are exposed to air. Enzymatic browning by polyphenoloxidase (PPO, EC 1.14.18.1) causes color and flavor degradation of foods [1,2,3,4,5]. Mechanical injuries to whole fruits/vegetables during postharvest handling and storage, and in processing whole fruits into juices, purees/smoothies, and cut products (e.g., cubes, slices, etc.) promotes the enzymatic reaction, with subsequent economic losses to the producers or manufacturers. The much appreciated fresh-cut minimally processed fruit and vegetable products are a class of foods also very susceptible to PPO browning [6]. PPO is an endogenous enzyme, which is naturally present in plant tissues, and it has also been referred to as polyphenol oxidase, catechol oxidase, tyrosinase, phenolase, catecholase, and o-diphenol oxidase. It is an oxidoreductase-copper-containing metalloprotein, which catalyses the degradation of phenolic fruit constituents to o-quinones in the presence of oxygen. The resulting o-quinone will subsequently polymerize with other o-quinone, protein or amino acids, producing undesirable brown compounds [7,8]. The activity of the enzyme can be reduced in the presence of antioxidants. Enzymatic browning can be inhibited by agents naturally present in foods such as lemon, onion, pineapple, grape, and wine [9], or otherwise through the inactivation of the enzyme by physical pasteurization methods as described in the following sections.

### 1.2. Thermal Pasteurization and Kinetics of PPO Enzyme Inactivation in Foods

Pasteurization is primarily used to reduce pathogenic- and/or spoilage-vegetative-microorganisms in foods and beverages, increasing their safety and shelf life [10,11,12,13]. However, as endogenous enzymes can also spoil the food and be more resistant than microorganisms, they are also used as pasteurization targets [14,15]. The conventional thermal pasteurization and blanching operations in the range of 80 to 100 °C inactivate the PPO enzyme contained inside the fruit and vegetable products, according to a linear pattern of the natural logarithm of PPO residual activity vs. processing time [16] (Equation (1)). In Equation (1), *A* and *A*_0_ are enzyme activities after and before processing, respectively. *A/A*_0_ is the enzyme residual activity (RA) after processing for a specific time *t* (min), and *k_T_* is the first order inactivation rate at temperature *T* (min^−1^).

(1) $$\frac{A}{A_{0}}=\exp\left(-k_{T}t\right)\;\mathrm{or}\;\ln\frac{A}{A_{0}}=-k_{T}t$$

Higher inactivation rates mean lower enzyme resistance. The Arrhenius equation describes the temperature dependence of the PPO inactivation rates (Equation (2)):

(2) $$\ln\left(k_{T}\right)=\ln\left(C\right)-\frac{E_{a}}{R}\left(\frac{1}{T}\right)$$

where *T* is the temperature (K), *E_a_* is the activation energy (kJ/mol), *R* is the universal gas constant (8.314 J/(mol·K)) and *C* is Arrhenius constant.

### 1.3. Ultrasound and High Pressure Processing Pasteurization Technologies and Kinetics of PPO Enzyme Inactivation

Due to the demand for fresh and minimally processed, preservative-free fruit/vegetable products, the emerging non-thermal food preservation technologies alone and combined with mild heat, and their effect on PPO inactivation has been investigated [5]. 

#### 1.3.1. Power Ultrasound

Power ultrasound (US) consists of sonic waves with frequencies higher than human ear audible sound (20–24 kHz). Power ultrasound or high-intensity ultrasound uses lower frequencies than medical applications, being characterized by sound intensity or acoustic intensity ranging between 10 to 1000 W/cm^2^ [17]. The sonication generates bubbles in liquid food as the wave energy propagates, a phenomenon known as acoustic cavitation. The formation and collapse of tiny bubbles can occur in few microseconds, and the cavitation increases with acoustic energy(power). The specific acoustic energy/power (W/g) or volumetric acoustic energy/power density (AED/AEP, in W/mL of processed liquid sample) quantify the energy requirements of the process for a given volume of treated food. Higher sound intensity/energy/power also increases the mechanical and sonochemical effects [15]. Ultrasound is known to break up proteins, starches and other large biopolymers such as enzymes, and can affect protein and enzyme functionality [18]. The damage to protein structure by ultrasound is desirable since it results in enzyme inactivation. Changes in enzyme biological activity are due to changes in the folding of the proteins, the secondary and tertiary structures of the enzyme. Under these extreme changes, hydrogen bonds and van der Waals bonding in the enzyme polypeptide chains can be broken down [19], resulting in the loss of enzyme activity [20]. The sonication processing conditions can cause partial or total inactivation of enzyme activity, depending on the type and the fruit/vegetable source of the enzyme. Thermosonication (TS) or heat-assisted ultrasound—the simultaneous application of US with heat—can be used to increase the efficiency of the process. The effect of ultrasound treatment on PPO depends on ultrasonic intensity (acoustic energy), duration of process (*t*), temperature (*T*), food/beverage matrix pH and ionic strength. A review by Silva and Sulaiman (2017) and O’Donnell et al. (2010) demonstrated that inactivation by US and TS also follow first-order reaction kinetics, similar to thermal inactivation alone [15,21]. In addition, it was concluded that TS is more efficient than ultrasound alone, in the inactivation of PPO across different fruit/vegetable juices and purees.

#### 1.3.2. High Pressure Processing

High pressure processing (HPP) or high hydrostatic pressure (HHP) is a cold pasteurization technology by which vacuum packed food products are introduced to a vessel containing a pressure transmitting liquid (usually water), and submitted to a high level of isostatic pressure (300–600 MPa). The nutritional and sensory characteristics of the original fresh fruit/vegetables, thus are better retained [22]. Several studies demonstrated that room temperature HPP has limited effectiveness towards the inactivation of the PPO enzyme [5,23]. Therefore, the combination of HPP with mild heat (60–90 °C), also referred to as high pressure thermal processing (HPTP), heat-assisted HPP or HPP-thermal, has been investigated for more effective enzyme inactivation [24]. HPP typically breaks non-covalent bonds (e.g., hydrogen bonds within the enzymes), but does not break covalent bonds (e.g., peptide bonds within the enzymes) [25]. Generally, it is expected that higher enzyme inactivation or lower enzyme residual activity (RA) will result when higher *T* (temperature for HPTP), *P* (pressure) and longer processes (*t*, time) are used for processing. Non-linear inactivation using a first order biphasic model with two rates (*k_L_* and *k_S_*) was previously used to model PPO inactivation by HPTP (Sulaiman et al., 2015) [24] (Equation (3)). *A_L_* and *A_S_* are activities of the labile and stable fractions, respectively, and *k_L_* and *k_S_* are the inactivation rate constants of labile and stable fractions of the enzyme, respectively.

(3) $$\frac{A}{A_{0}}=\frac{A_{S}}{A_{0}}\;\exp\left(-k_{L}t\right)+\frac{A_{S}}{A_{0}}\;\exp\left(-k_{S}t\right)\;$$

Other HPP-thermal studies carried out with lychee, sapodilla and Packham pear used the first order kinetic model (Equations (1) and (2)) [26].

In the present study, kinetic data of PPO enzyme inactivation in pear, apple, and strawberry purees by TS, HPTP, and thermal processing previously generated by Sulaiman et al. (2015) [16,24] were used with the following purposes: (i) to compare the efficiency of PPO inactivation by three different pasteurization technologies; (ii) to compare the resistance of PPOs across three different fruit purees.

## 2. Material and Methods

### 2.1. Fruits and Properties

Locally sourced ripe pear, apple, and strawberries were used for the inactivation kinetic experiments [16,24]. The fruits were peeled and cored (apple and pear), cut into smaller pieces, and blended using a commercial blender. Table 1 shows the fruits’ cultivars and some properties of the fruit purees. For thermal and HPTP treatments, 20 g of each fruit puree was packed in 150 mm × 105 mm food grade retort pouches (Cas-Pak, New Zealand). Fruit thermal conduction was minimized by packing a small size fruit sample in a large surface area pouch, thus no temperature distribution occurred, and fruit temperature could be considered uniform inside the bag. The packed samples were stored at −70 °C and thawed in a commercial refrigerator overnight before treatment. At least two replicates of packed samples were processed for each processing condition as described in the following section.

### 2.2. Fruit Purees Processing

After the treatments, the samples were immediately cooled in an ice-water bath before the enzyme extraction. The enzyme activity was determined for a raw, unprocessed sample across various temperatures (*A*_0_), as well as a processed sample (*A*) using the procedure described by Sulaiman et al. [27,28]. The average enzyme residual activity ± standard deviation (RA = residual activity = *A/A*_0_) was calculated and kinetics were modeled. 

#### 2.2.1. Thermosonication

The procedure for thermosonication and generation of residual activity kinetic data followed the procedure described by Sulaiman et al. (2015) [16]. Ultrasound processing was carried out using the UP200S (24 kHz) Hielscher Ultrasound GmbH (made in Germany) coupled with a 3 mm diameter sonotrode, which was submerged in 25 g of puree contained within a plastic cup. The equipment was set in a continuous mode of energy supply at 210 µm amplitude. The specific acoustic power was estimated from the ultrasound intensity specifications of the equipment manual for the 3 mm sonotrode. The total power applied (32.5 W) was determined from the product of the sonotrode area (0.0707 cm^2^) by the ultrasound intensity at maximum amplitude (460 W/cm^2^), whereas the specific acoustic power of 1.3 W/g was calculated from the ratio of the power and the mass of fruit puree treated (25 g). The temperature of the fruit puree was monitored with a thermocouple. The plastic cup containing the fruit puree sample was placed in a circulated thermostatic water bath which was set at a lower pre-treatment temperature than the TS treatment temperature, to account for natural temperature increase during TS due to heat dissipation. When the sample reached the designated internal temperature, the ultrasound was switched on and TS treatment time-counting began. Due to the increase in the temperature during the process, the average temperature during TS was considered as the treatment temperature. The enzyme inactivation rates at 71 °C (for the 3 fruits) and 57 °C (for strawberry) were estimated by linear regression (Equation (2)) from the first order rates at various temperatures between 33 and 72 °C as well as the activation energies previously determined for the three fruits [16]. 

#### 2.2.2. HPTP

The procedure for heat-assisted high-pressure processing treatments was described in detail previously by Sulaiman et al. (2015) [24]. Industrial units usually operate at room temperature and a maximum pressure of around 600 MPa. However, in this study HPP combined with heat (HPP-thermal or HPTP) was used for increased efficiency in terms of enzyme inactivation. Packed fruit puree samples were processed using the Avure 2L-700 HPP Laboratory Food Processing System (Serial No. 101130, USA) containing distilled water as the pressure medium in the treatment chamber. The HPP chamber was equipped with a thermocouple to register the temperature during the HPP cycle. This unit can operate at up to 600 MPa of pressure and at moderate temperatures. At the end of the constant pressure phase, the release of the pressure caused an instantaneous decompression. The HPTP pressure (600 MPa) and temperature (71 °C) selected for this study were the maximum allowed by the HPP equipment. Packed fruit puree samples were pre-heated before pressurization so that the average temperatures during the constant pressure phase of the HPP cycle were 57 °C (for strawberry) and 71 °C (pear, apple, strawberry). The pressure-temperature-time processing conditions refer to the constant pressure phase of the HPP cycle. The total pressure increase took less than 2 min. As kinetics is non-linear, a first order biphasic model with two inactivation rates, was fitted to the residual activity vs. time data (Equation (3)). 

#### 2.2.3. Thermal Processing

Thermal inactivation details of the experiments carried out are described by Sulaiman et al. (2015) [16]. The puree samples were fully submerged in a thermostatic water bath at various temperatures between 50 and 85 °C depending on the PPO fruit origin and resistance (W28 Grant Instruments Ltd., Cambridge, England) for pre-specified processing times. After processing, the samples were immediately cooled in an ice-water bath before enzyme extraction and activity analysis. The enzyme inactivation rate at 71 °C was estimated by linear regression (Equation (2)) from the first order inactivation rates at various temperatures and activation energies, as previously determined for the three fruits [16]. In addition, for strawberry the inactivation rate (k) was also estimated for 57 °C. 

### 2.3. Kinetic Charts Generated 

**Figure 1:**
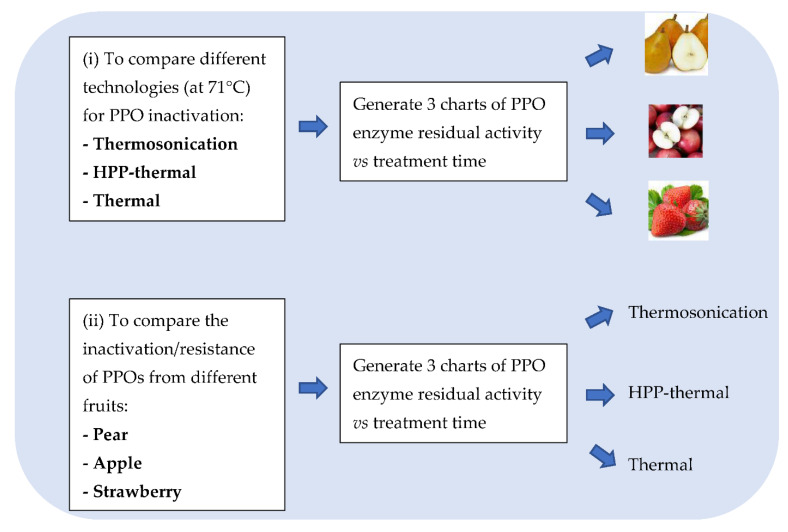
Charts plotted and study objectives.

For the first objective of comparing the efficiency of the 3 technologies in terms of PPO enzyme inactivation, 3 charts at 71 °C for pear, apple and strawberry PPOs were plotted (Figure 1). An additional chart at 57 °C was plotted for strawberry to assess the effect of temperature on the PPO inactivation kinetics. The second objective of this study was to compare the resistance of PPOs from different fruits/cultivars. One chart for each technology was replotted to study PPO resistance to TS, HPP-thermal and thermal technologies (Figure 1). 

## 3. Comparison of Three Pasteurization Technologies for the Inactivation of PPO in Fruit Puree

**Figure 2:**
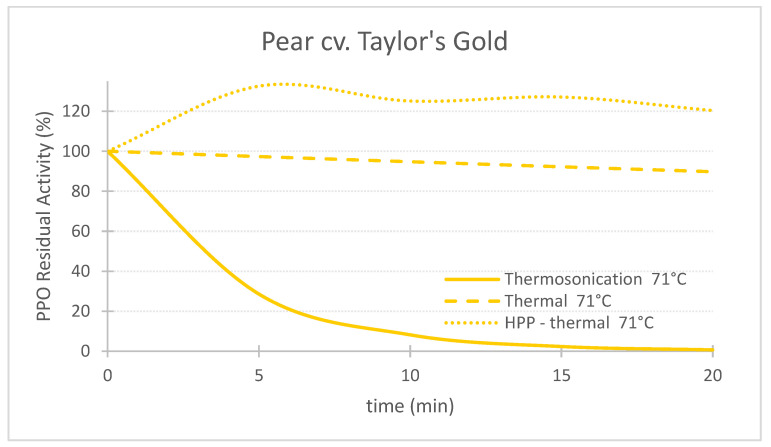
Comparing the effect of thermosonication (TS, 1.3 W/g—71 °C), heat assisted high pressure processing (HPTP, 600 MPa—71 °C) and thermal process alone (71 °C) on polyphenoloxidase enzyme residual activity during treatment time for ‘Taylor’s Gold’ pear puree.

**Figure 3:**
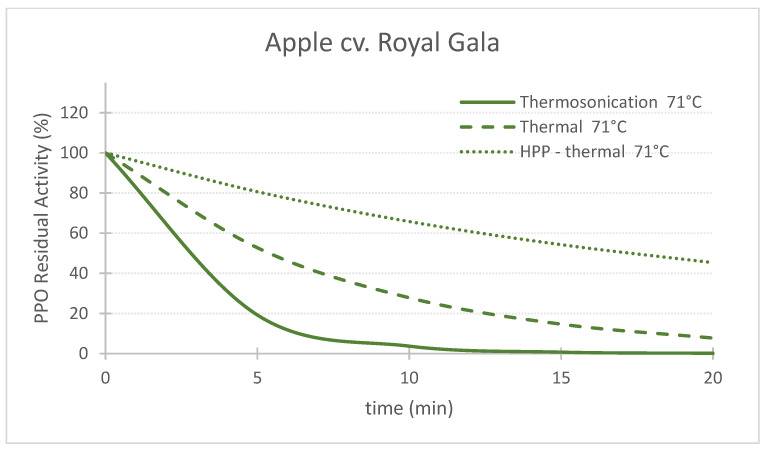
Comparing the effect of thermosonication (TS, 1.3 W/g—71 °C), heat-assisted high pressure processing (HPTP, 600 MPa—71 °C) and thermal process alone (71 °C) on polyphenoloxidase enzyme residual activity during treatment time for ‘Royal Gala’ apple puree.

**Figure 4:**
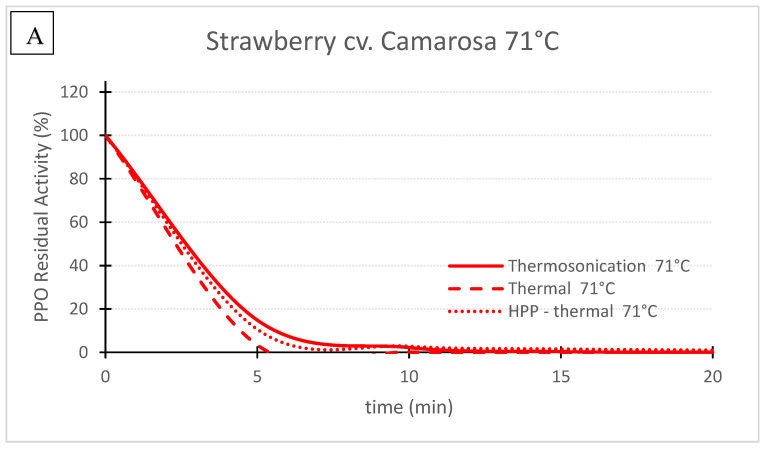

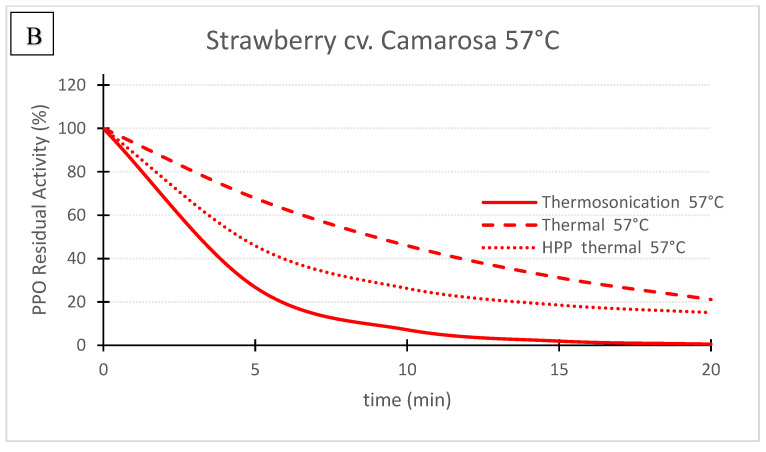
‘Camarosa’ strawberry polyphenoloxidase residual activity after thermosonication (TS, 1.3 W/g), heat assisted high pressure processing (HPTP, 600 MPa) and thermal treatment alone at 71 °C (**A**) and 57 °C (**B**).

**Figure 5:**
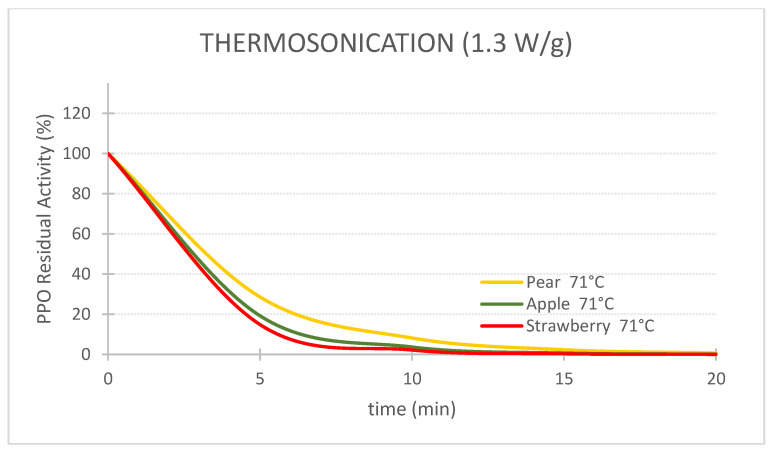
Comparison of the inactivation of polyphenoloxidase across three different fruits by thermosonication (1.3 W/g, 71 °C).

**Figure 6:**
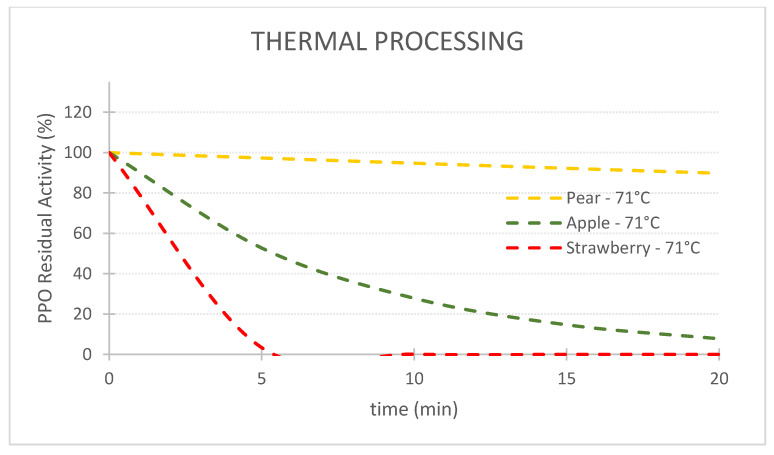
Comparison of the inactivation of polyphenoloxidase in three different fruits by thermal processing alone (71 °C).

**Figure 7:**
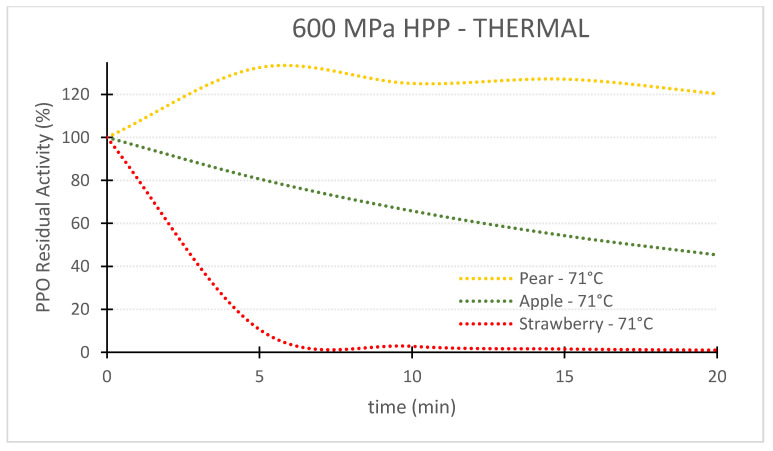
Comparison of the inactivation of polyphenoloxidase in three different fruits by high pressure thermal processing (HPTP, 600 MPa, 71 °C).

Table 2 shows the inactivation rates (*k*) used to generate the charts of PPO Residual Activity (RA) vs. time presented in the Figure 2, Figure 3, Figure 4, Figure 5, Figure 6 and Figure 7. In general, TS rates were the highest, indicating a quicker inactivation by TS compared with thermal and HPP-thermal processes. HPP-thermal presented the lowest inactivation rates (stable fraction of the enzyme), meaning more time is needed for the same enzyme inactivation. 

Figure 2, Figure 3 and Figure 4 compare the effect of the three technologies on the PPO enzyme at 71 °C for pear, apple, and strawberry, respectively. Figure 4 also shows the effect of the three technologies on the PPO enzyme in strawberry at a lower treatment temperature of 57 °C. TS was the best technology for inactivating pear and apple PPO enzymes at 71 °C (Figure 2 and Figure 3), and strawberry PPO at 57 °C (Figure 4). Furthermore, thermal treatment alone was better than HPP-71°thermal for all the three fruits (Figure 2, Figure 3 and Figure 4). However, at a lower temperature (57 °C), HPP-thermal caused higher inactivation of strawberry PPO than thermal treatment alone (Figure 4B). The residual activity results obtained at 71 °C for strawberry puree demonstrated that at this temperature there is no added benefit of using HPP-thermal compared with exclusively thermal processing, contradicting the results obtained at 57 °C. 

With respect to pear PPO (Figure 2), only TS was efficient for PPO inactivation, requiring 10, 15 and 20 min at 71 °C to reduce RA from 100% to 8.2%, 2.3% and 0.7%, respectively. On the contrary, the exclusive use of thermal processing at the same temperature barely had an effect on the PPO enzyme, still exhibiting 89.8% RA after 20 min. Furthermore, HPP-thermal activated the enzyme, expressed by a RA higher than 100%, for 20 min treatment, 120.3% RA activity was obtained, corresponding to 20.3% enzyme activation. Therefore, HPP is not recommended for preservation of this particular variety of pear. 

Figure 3 and Figure 4 show that this activation effect was not registered in apple or strawberry enzymes, as the 3 technologies reduced PPO activity with processing time. The activation of enzymes HPP can occur [29] with HPP treatment, at room temperature in particular, as HPP treatment changes the conformation of the protein enzyme [5]. Enzyme activation by HPP was also observed for Bartlett pear slices at 400 MPa [30], Boskoop apple juice at 400 MPa [31], Amasaya apple juice at 450 MPa [32], sweet potato puree [33] and whole mushroom treated at 600 MPa [34]. Activation of PPO enzyme in Nashi pear and Royal Gala apple purees HPP treated at 600 MPa was also registered [24]. However, in the last study, when combining 600 MPa with a temperature of 62 °C, inactivation was registered for both fruit cultivars. 

The comparison of RA of apple PPO after 10 min processing at 71 °C demonstrated the supremacy of TS over the other technologies, as RA were 3.7%, 27.8% and 65.8% for TS, thermal and HPP-thermal, respectively (Figure 3). 

Figure 4 compares the inactivation of strawberry PPO at 57 and 71 °C by the 3 technologies. For 71 °C the kinetics of PPO inactivation and RA registered for the 3 technologies were very similar (Figure 4A). For example, after 10 min, 2.2%, 0.1% and 2.8% were obtained for TS, thermal and HPP-thermal treatments, respectively. At 57 °C all the inactivation rates were lower than at 71 °C, demonstrating the great effect of temperature alone or combined with other pasteurization technologies (Table 2), whereas at 57 °C, RA after 10 min were 7.1%, 26.1% and 45.9% for TS, HPP-thermal and thermal processing alone, respectively (Figure 4B). At 71 °C, residual activities below 3% were obtained for the same treatment time with similar inactivation history for the 3 pasteurization methods (Figure 4A). 

## 4. Kinetics and Resistance to Inactivation of Pear, Apple and Strawberry PPOs in Fruit Purees

Figure 5, Figure 6 and Figure 7 compare the PPO residual activity pear, apple and strawberry (and their relative resistance) during TS, HPP-thermal and thermal treatments, respectively. TS at 71 °C revealed efficient and similar inactivation of the three fruits’ enzymes (Figure 5). Therefore, the resistance of the three fruits’ PPOs to TS is similar. For example, after 10 min, RA of 8.2%, 3.7% and 2.2% were obtained for pear, apple and strawberry PPO, respectively. 

When applying thermal processing alone or HPP-thermal at the same temperature, huge differences in the RA of the three fruits’ PPOs were observed, with pear PPO presenting more resistance, followed by apple PPO and lastly strawberry PPO. RA of 94.8%, 27.8% and 0.1% were registered after a 10 min thermal treatment at 71 °C for pear, apple, and strawberry PPOs, respectively (Figure 6). 

For 600 MPa—71 °C—10 min 125.1% for pear PPO RA (25% activation of PPO enzyme), 65.8% for apple PPO RA and 2.8% for strawberry PPO RA were obtained (Figure 7), following the same pattern of resistance observed with the other technologies. A review by Silva and Sulaiman (2019) [5] also showed that enzymes from different fruits/vegetables, and within the same species from different varieties have different conformations, which confers different resistances to inactivation.

## 5. Conclusions

The use of non-thermal technologies allows the reduction in treatment time or temperature, resulting in a food product of higher quality [35,36]. Among the three technologies investigated, TS was the best for inactivating endogenous PPO enzyme in fruits, followed by thermal processing. High pressure processing combined with heat at 71 °C was less efficient than TS and thermal treatment alone, and in the case of pear, undesirable enzyme activation was registered. Therefore, this technology might not be appropriate for certain foods containing PPO enzyme, as it can promote enzymatic browning. As expected, increases in the processing temperature subsequently increase the rates of enzyme inactivation for TS, thermal and HPP-thermal technologies. The resistance of the three fruits PPOs presents the same pattern, as pear PPO was the most resistant enzyme to the three technologies employed, followed by apple PPO and lastly strawberry PPO. This study demonstrated how variable the inactivation or resistance of PPOs from different fruit origins can be, including the fruit species, and within the same species, the fruit cultivar.

## Figures and Tables

**Table 1:** Pear, apple and strawberry cultivars and puree properties.

Fruit Common and Scientific Name	Cultivar or Variety	Total Soluble Solids (°Brix)	pH
Pear *Pyrus communis*	Taylor’s Gold	16.74 ± 0.34	4.55 ± 0.17
Apple *Malus domestica*	Royal Gala	10.87 ± 0.12	4.01 ± 0.12
Strawberry *Fragaria ananassa*	Camarosa	9.30 ± 0.10	3.31 ± 0.15

**Table 2:** First order enzyme inactivation rates for thermosonication (TS) and thermal inactivation, and first order biphasic enzyme inactivation rates for heat assisted high pressure processing (HPP-thermal) of Taylor’s pear, Royal Gala apple and Camarosa strawberry purees *.

		**TS** **(1.3 W/g)**	Thermal	HPP—Thermal (600 MPa)	
	**Kinetic Model**	**First Order**	First Order	First Order Biphasic	
**Fruit**	**Temperature (°C)**	$k_{T}$ **(min^−1^)**	$k_{T}$ **(min^−1^)**	$k_{L}$ **(min^−1^)**	$k_{S}$ **(min^−1^)**
Pear	71	0.25	0.0054	No inactivation	No inactivation
Apple	71	0.33	0.13	0.061	0.018
Strawberry	71	0.38	0.68	0.51	0.081
Strawberry	57	0.26	0.078	0.21	0.018

* The first order rates (*k_T_*) at 71 °C and 57 °C were estimated using the original model *E_a_* (Sulaiman et al., 2015) [16]; the first order biphasic rates for stable (*k_S_*) and labile (*k_L_*) fractions were taken from Sulaiman et al. (2015) [24].

## Data Availability

The date are not publicly available.
